# SCALS: a fourth-generation study of assisted living technologies in their organisational, social, political and policy context

**DOI:** 10.1136/bmjopen-2015-010208

**Published:** 2016-02-15

**Authors:** Trisha Greenhalgh, Sara Shaw, Joe Wherton, Gemma Hughes, Jenni Lynch, Christine A'Court, Sue Hinder, Nick Fahy, Emma Byrne, Alexander Finlayson, Tom Sorell, Rob Procter, Rob Stones

**Affiliations:** 1 Nuffield Department of Primary Care Health Sciences, University of Oxford, Oxford, UK; 2 Blizard Institute, Barts and the London School of Medicine and Dentistry, London, UK; 3 Department of Politics and International Studies, University of Warwick, Warwick, UK; 4 Department of Computer Science, University of Warwick, Warwick, UK; 5 Sociology and Criminology Department, University of Western Sydney, Sydney, Australia

**Keywords:** telehealth, telecare, new models of care, co-design, organisational case study, structuration

## Abstract

**Introduction:**

Research to date into assisted living technologies broadly consists of 3 generations: technical design, experimental trials and qualitative studies of the patient experience. We describe a fourth-generation paradigm: studies of assisted living technologies in their organisational, social, political and policy context. Fourth-generation studies are necessarily organic and emergent; they view technology as part of a dynamic, networked and potentially unstable system. They use co-design methods to generate and stabilise local solutions, taking account of context.

**Methods and analysis:**

SCALS (Studies in Co-creating Assisted Living Solutions) consists (currently) of 5 organisational case studies, each an English health or social care organisation striving to introduce technology-supported services to support independent living in people with health and/or social care needs. Treating these cases as complex systems, we seek to explore interdependencies, emergence and conflict. We employ a co-design approach informed by the principles of action research to help participating organisations establish, refine and evaluate their service. To that end, we are conducting in-depth ethnographic studies of people's experience of assisted living technologies (micro level), embedded in evolving organisational case studies that use interviews, ethnography and document analysis (meso level), and exploring the wider national and international context for assisted living technologies and policy (macro level). Data will be analysed using a sociotechnical framework developed from structuration theory.

**Ethics and dissemination:**

Research ethics approval for the first 4 case studies has been granted. An important outcome will be lessons learned from individual co-design case studies. We will document the studies’ credibility and rigour, and assess the transferability of findings to other settings while also recognising unique aspects of the contexts in which they were generated. Academic outputs will include a cross-case analysis and progress in theory and method of fourth-generation assisted living technology research. We will produce practical guidance for organisations, policymakers, designers and service users.

**Strengths and limitations of this study**

- Introduces and applies the ‘fourth generation’ approach to the study of assisted living technologies in organisational, social and political context.
- Aims to collect rich qualitative data at three levels: micro (the patient experience), meso (organisational routines and processes) and macro (policy and industry context).
- Analysis views the technology as part of a dynamic, networked and unstable system.
- Includes an action research component to generate local solutions and produce cross-case practical learning.
- Designed to highlight situated behaviours and transferable insights to comparable settings.
- Not designed to generate an ‘effect size’ or formulaic service solution.

## Introduction

### Background

It is more than 20 years since Mark Weiser first mooted the idea of the ‘smart home’, in which computer technologies, built unobtrusively into the domestic environment, would improve people's quality of life in numerous ways (including security, energy consumption, leisure opportunities, health and well-being).^1^ A generation of research into home-based assisted living technologies—which include telecare (alarms and sensors that detect emergencies such as falls or environmental hazards such as smoke or carbon monoxide) and telehealth (remote monitoring of biomedical markers such as blood pressure, weight or oxygen levels)—has produced many prototypes, along with predictions of improved health status, patient empowerment and a better, safer, more integrated and more efficient health service.^2–4^

Despite this research (and, some would argue, with the single exception of pendant alarms), assisted living technologies have been characterised by limited uptake, high rates of abandonment and numerous challenges (economic, operational, technical, ethical, clinical) when attempts have been made to embed them into routine health and social care services.^3–14^ The policymakers’ prediction in 2012 that ‘3 million lives’ would be saved through assistive technologies developed through a ‘concordat’ between government and the technology industry^15^ has yet to materialise. In short, these technologies represent a classic—though complex—case study of the *non-adoption* of technological innovations.

Research to date into assisted living technologies can be divided, broadly speaking, into three overlapping ‘generations’. First came technical design: studies undertaken largely by computer scientists to develop technologies and demonstrate proof of concept (ie, that the technologies ‘worked’ in controlled conditions).^7^ ^8^ ^16^ Second came experiments—especially randomised controlled trials, designed and conducted mostly by doctors (who viewed the clinical trial as the most robust way to test anything that was offered to a patient). Participants were typically assigned to an intervention (‘technology plus usual care’) or control (‘usual care’) arm and followed up against predefined outcome measures (such as health status, mortality, use of services and cost).^5^ ^14^ Notably, the large Whole System Demonstrator trial in the UK showed that participants randomised to telehealth or telecare had significantly fewer hospital admissions and lower mortality in the subsequent year—but that these benefits were achieved at a cost per quality-adjusted life year (£88 000 for healthcare, and £297 000 for social care) that most local commissioners would deem unaffordable.^11^ ^17–19^

The third generation of research into assisted living comprised qualitative studies of the patient experience. Designed and led mainly by social scientists, nurses and professions allied to medicine, they highlighted the uniqueness of individual needs and aspirations; the importance of a careful assessment of the social and material context into which technologies would be introduced; the awkwardness of standardised solutions; the potentially negative impacts (eg, social isolation) of even ‘successful’ assistive technologies; and the crucial role of family and carers in adapting and supporting installed technologies to maximise fitness for purpose as the person's health status, and circumstances changed over time.^20–27^

All these approaches have their place, but the limits of proof-of-concept technical design, experimental trials and small-scale qualitative studies have become evident. The range of available technologies is vast, rapidly evolving, and defies taxonomy.^13^ Today's published research always relates to yesterday's version of the technology. Research into one technology in one context will not predict the effectiveness or acceptability of another technology in a different context. There is thus a sense of ‘sorcerer's apprentice’—a field that is outstripping the capacity of researchers to understand and test it.^28^ Neither assisted living technologies nor the people who use them can be studied effectively in isolation from the complex sociotechnical system in which they are (perhaps imperfectly) embedded. In particular, when randomised trial designs are used to ‘control for’ the multiple organisational, social, cultural and political influences on which this embedding depends, the external validity of any effect size becomes questionable.^29^ Technical descriptions and trials of ‘technology on’ versus ‘technology off’ reflect technological determinism (the notion that the introduction of a technology can *determine* a particular outcome)—a perspective that has long been discredited by sociologists of science.^30^ ^31^ Technologies may create opportunities but they do not, in and of themselves, *cause* personal, organisational or social change.^32^ Qualitative studies documenting the design–reality gap on a case-by-case basis may inform, but they do not produce, solutions to this gap.

For all these reasons, it is time for a paradigm shift. We propose a fourth generation of assisted living technology research, with five key characteristics. First, unlike the previous three generations (which, with some rare exceptions described below, were more or less unidisciplinary traditions in computer science, biomedicine and social science, respectively), the fourth generation paradigm is interdisciplinary—drawing on, and synthesising, these previous perspectives along with input from (among other disciplines) management studies, bioethics and political science.

Second, it *embraces complexity*. It acknowledges, and seeks to illuminate, the organisational, social, political and policy context in which assisted living technologies are developed, introduced, supported and used (or not). More specifically, it views people and technologies as linked in dynamic, networked and potentially unstable systems made up of multiple interacting stakeholders.

Third, the new paradigm is *recursive*—that is, it views human decisions and actions (‘micro’) as both influenced by, and influencing, the wider context of family and organisation (‘meso’), and of society and system (‘macro’). Thus, for example, the development and introduction of an assistive technology is seen as intimately and reciprocally entwined with the development of the health or social care service, the particular lay and professional networks that support the technology's use, and with local, national and transnational policy on technological innovation and assisted living.

Fourth, the new paradigm is *ecological*. It rejects, for example, the notion of specific solutions that are unproblematically transferable elsewhere. Solutions must be (at least partly) locally grown and collectively owned. The ecological paradigm also problematises the idea of a linear link between (upstream) research and (subsequent) implementation of findings in favour of an emergent, collaborative approach in which solutions are co-designed by multistakeholder groups that include researchers, technical designers, care commissioners, health and social care professionals and end users (hence, implementation occurs in parallel with research, not after it).^33^

Finally, the new paradigm is (in the sociological sense) *critical*. Because different stakeholders have competing interests, the complex systems on which assisted living solutions depend are potentially sites of both overt and covert power struggles. A rigorous analysis of the adoption, non-adoption and abandonment of assisted living technologies in health and social care must explore whose interests are served by different arrangements and eventualities. In the co-design process, much will depend on both formal contexts (especially how stakeholders are governed and regulated) and informal ones (histories, interpersonal relationships, etc).

We do not claim to be the first to propose an interdisciplinary, complex systems perspective with a recursive, ecological and critical lens for the study of technology programmes. On the contrary, such an approach is (broadly speaking) shared by a number of established traditions that draw variously on management theory,^30^ critical realism,^34^ actor-network theory^35^ and structuration theory.^36^

For many years, these traditions focused on fields other than healthcare. Recently, however, social scientists have begun to draw eclectically on them in what has become known as ‘sociomaterial studies’ of healthcare technologies.^37–41^ May's normalisation process theory can also be thought of as addressing the recursive relationship between technologies, their users and the organisational and social context.^42^ Nicolini has applied practice theory to study the complex, embodied, interactive and materially mediated nature of knowing in telemedicine.^43^ Maniatopoulos *et al*^44^ drew on the notion of ‘field of practices’ to examine how adoption of a new diagnostic technology for breast cancer was subject to spatially and temporally distributed reconfigurations across a multilevel set of practices, from macro (policy) to the micro (individual action). Hollnagel *et al*^45^ have developed the concept of ‘resilience’ in healthcare organisations, focusing (instead of accounting for failure) on the study of active and adaptive efforts of organisational members that contribute to things going right. Similar conclusions are to be found in studies in the dependable systems literature.^46^ We have previously combined a version of structuration theory with selected elements of actor-network theory to theorise the complex, rapidly changing, and heavily regulated setting of national IT programmes in UK healthcare.^47^

All these approaches might be considered ‘fourth generation’ in that they address complexity and are recursive, ecological and critical. We seek not merely to extend existing work but also to mainstream fourth-generation approaches by linking their theoretical roots (in the social sciences) with practical application and impact (in health services development). In the remainder of this paper, we describe how we will adapt our structuration theory approach to study the development, application and use of assisted living technologies as part of evolving health and care services in the real world.

### Our assisted living research to date

The SCALS (Studies in Co-creating Assisted Living Solutions) programme described in this paper builds on previous work by our team, especially the ATHENE (Assistive Technologies for Healthy Living in Elders—Needs Assessment by Ethnography) study, which was funded by the Technology Strategy Board from 2010 to 2013,^2^ ^48–55^ as well as on a recent PhD study.^56^ These studies were predominantly qualitative and ethnographic but they also attempted to explore the organisational and system context of individual examples of technology use and non-use.

The ATHENE study demonstrated the crucial importance of *bricolage*—needs-focused adaptation and customisation of technologies for the person with multimorbidity by someone who knows and cares for them.^49^ ^51^ It fed into a co-design phase in which technology users were brought together with industry, health and social care services to inform refinements to design.^33^ One output from ATHENE was a new set of standards and principles for telehealth and telecare services, known as the ‘ARCHIE’ framework: telehealth and telecare should be *anchored* in what matters to the patient or client; *realistic* about the natural history of illness and ageing; *co-creative* (evolving and adapting solutions with users); *human* (supported through interpersonal relationships and social networks); *integrated* through attention to mutual awareness and knowledge sharing; and *evaluated* to drive system learning.^48^

Our work to date has shown that current UK arrangements for health and social care practitioners to assess people for assisted living technologies and supporting them to use these are suboptimal. With some rare exceptions, they are predicated on a plug-and-play model of technology, a customer-contractor model of assessment and installation, and an emphasis on ‘innovation’ (ie, incentivising industry to produce new technologies) rather than on supporting the adaptation, recombination and ongoing support of existing technologies.^2^ ^51^ ^53^ This is occurring in the context of a strong policy pressure to implement technologies ‘at scale’ rather than produce solutions more slowly for a smaller sample of individuals so as to maximise system learning. Our conclusion from our previous work was that

Not only have we not come up with a specification for a technology that will ‘fix’ the challenges of telehealth and telecare provision; we have demonstrated that no such technological fix can ever be developed. The solutions we propose […] are orders of magnitude more difficult to deliver, since they demand far-reaching changes in the organisation and delivery of services, the way health and care organisations purchase technologies, the way staff from these organisations work together on the ground, and the level of ongoing commitment by all players that will be needed to maintain an assisted living solution once it has been developed. 48

In summary, work by ourselves and others to date has demonstrated that ‘calm’, scalable technological solutions to the challenges of an ageing society are a modernist myth. In reality, solutions that work in practice will always be an effortful sociotechnical accomplishment across multiple organisational and personal boundaries that is characterised by competing interests and inherent conflicts. While we acknowledge the extraordinary success of technological developments (eg, Apple's iPhone, iPad, etc) when promoted to private citizens who act as individual adopters, early data from SCALS shows that these same technologies are not rapid or unproblematic drivers of change in the heavily institutionalised environment of healthcare (their introduction and use, for example, requires board approvals, an organisation-wide supplier contract, standard operating and information governance procedures, a recurrent budget line for technical support, a staff training programme and usage monitoring). In other words, even the most elegantly designed technologies must be considered as part of a wider sociotechnical ensemble that may strongly influence adoption and subsequent adaptation in use. As Barley observed a generation ago, technologies in healthcare organisations are an ‘occasion for structuring’ (ie, they provide *opportunities* for change, but they do not *determine* change in any simple sense).^32^ It is time to advance the way we study how such sociotechnical structuring may occur (or fail to occur) locally, and how lessons can be gleaned and transferred from both successes and failures.

## Methods and analysis

### Aim

To study assisted living technologies in their organisational, social, political and policy context, using a complex systems approach that considers interdependencies, emergence and conflict.

### Objectives

1. To recruit a maximum variety sample of health and social care organisations seeking to improve services to older people with complex health and social care needs with the aid of technology.
2. To support these organisations in developing, delivering and evaluating their chosen service using an action research design, informed by ethnography and contextual analysis.
3. To generate wider empirical lessons about the introduction of technology-supported services in health and social care.
4. To build theory and method about fourth-generation research into assisted living.

### Research questions

1. How can we improve the development of assisted living technologies by and for people with multimorbidity and declining health?
2. How can we better promote the customisation and use of such technologies in the home and the community by individuals, their carers and support services?
3. How can we ensure that ethical and existential concerns (What matters to people? What are homes for? How should we live? What are society's responsibilities towards its sick?) inform and shape the development, introduction, adaptation and use of assisted living technologies?

### Overview of study design

**Figure 1:**
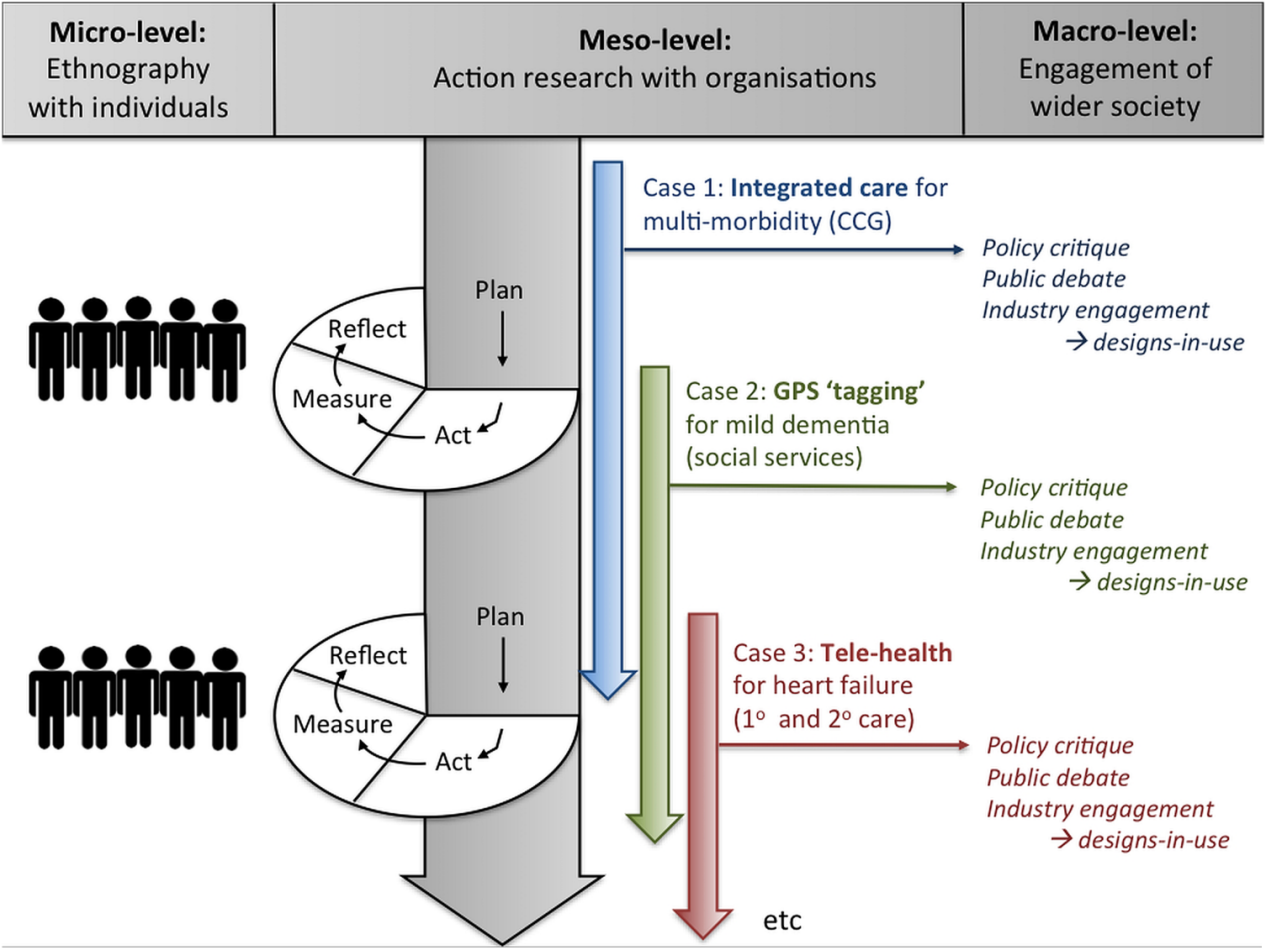
Diagram of the SCALS (Studies in Co-creating Assisted Living Solutions) programme (first three case studies shown).

The research has three linked components: micro (ethnographic studies of multimorbidity and ageing); meso (organisational and system change, including embedding of technologies) and macro (policy analysis, public debate and industry engagement). As in our previous ATHENE study, we will use lived experiences of real participants, studied using ethnographic methods in and around the home,^57^ as a key element of the programme. These micro (individual) case studies will be an integral part of, and used to inform, a set of evolving meso (organisational-level and system-level) case studies drawing on the principles of action research^58^ and experience-based co-design.^59^ The case studies, and an accompanying cross-case analysis and synthesis, will, in turn, both inform and be informed by a wider case study of national policy, industry strategy and prevailing public opinion on key policy issues, such as ageing, multimorbidity (including integrated care) and assisted living technologies. This study design is illustrated schematically in figure 1.

A crucial feature of the SCALS research programme is that it is technology agnostic (or more accurately, agnostic with respect to the value of particular technologies in specific circumstances). Aligning with others who have undertaken critical ethnography of technologies in the home,^21^ ^22^ ^26^ ^37^ ^60–66^ we deliberately seek to ‘de-centre’ technology and, instead, place at the centre of our analysis the person's lived experience of illness, the clinical and social care microsystems and the wider health and social care systems within which that experience is nested. Technologies may be a crucial component of that lived experience and those (micro)systems, but we analyse them as they emerge and are used as part of the system, not as freestanding objects of study with an ‘impact’ all of their own.

The SCALS case studies will explore further a key finding from the ATHENE study of the importance of pragmatic, needs-focused adaptation (‘bricolage’), which has since been replicated by others.^67^ Bricolage may involve acquiring new technologies—but it often consists mainly of ‘fiddling’ with legacy technologies (things already present in the home or passed on secondhand from friends or relatives) to make them work in new ways and/or for new purposes.^68^ Bricolage, as applied to living with multimorbidity, is an under-researched area. Our research focus is the *process of bricolage* and how it might fit with the personalisation of care packages (lay and professional), rather than any particular technology that gets produced.

### Theoretical/conceptual framework

Our work is grounded in phenomenological philosophy; it seeks to understand illness and ageing from the perspective of the individual.^21^ ^49^ ^69^ Importantly, the introduction and use of assistive technologies by health and social care professionals looking after patients and clients is an *ethical practice*, bound by professional codes of conduct (confidentiality, respect for autonomy, etc) and strongly linked to professional identity.^70^ ^71^ To link this with the organisational context of care and with wider society, we will apply Stones’ strong structuration theory, which conceptualises individuals’ *internal structures* (ie, their past experience, cultural background, knowledge, beliefs, values, perceptions and so on—akin to what Bourdieu called *habitus—*along with their ongoing, phenomenological experience of illness and ageing) as recursively linked to (ie, both shaping and shaped by) *external structures* of social norms, rules, laws, standards, policies and so on.^72^

As noted above, Greenhalgh and Stones have refined and applied strong structuration theory to study the relationship between individual agency and social structures in relation to self-management and the use of technologies in healthcare.^47^ ^69^ ^73^ What we have not done previously is focus this at the *meso* level to explore how social structures (cultural, regulatory, political) are inscribed in organisational forms and processes, and in the technologies that are supplied and supported by these organisations, to both create possibilities and limit what is possible for the human agents who work for, and/or are served by, those organisations.

The starting point in our meso-level case studies will be a ‘problem to be explored’ (referred to in strong structuration theory as an *explanandum*),^72^ identified through both ethnographic observation and staff interviews. This might well be identified as a set of current outcomes or patient/client experiences, which are deemed to be unsatisfactory. The notion of *retroduction* (ie, asking ‘what mechanisms and influences might explain…’) produced from within critical realism is useful here. For each explanandum, data will be gathered and discussions held to elucidate, and adjudicate, between possible mechanisms or processes that have brought this about. These mechanisms, and their interactions, will be empirically tracked by gathering further data as needed.

The purpose of this theorisation is to identify the *generative causes* of the unsatisfactory outcomes (or, in some cases, of satisfactory outcomes). One key data source will be the explanations and reflections of ‘proximate actors’—that is, of individual patients and clients, and (perhaps even more so) of front-line health and social care professionals, since the latter's perspective is informed by their close experiences with many patients/clients over the years—as they compare what would ‘ideally’ be in place with what is actually occurring on the ground. In the case of unsatisfactory outcomes, we would also be seeking possible *strategies* (eg, a change in policy) that would alter, replace, or counter some of the generative causes, so as to produce more satisfactory outcomes. It will be important here to explore the perceptions held by front-line professionals as to what are obdurate but potentially malleable obstacles to positive change, and to differentiate these from constraints that are considered to be entirely intractable.

The pressures and constraints within and among organisations that affect staff behaviour are complex and conflicting. Sometimes, a specific set of external, organisational or interorganisational pressures can be uppermost in people's consciousness, and strongly influence their judgments about how to behave. At other times, in relation to other tasks, it will be other parts of the contextual field that will loom largest. It is important to identify which pressures from the contextual field prevent proximate actors from carrying out their ideal practices with respect to which particular tasks.

The detail of this meso-level exploration will be developed further as the programme unfolds, and be the subject of a theoretical paper.

### Setting and context

The SCALS programme is recruiting health and social care organisations across England, selected to provide maximum variety in demographic, geographic, sociocultural and organisational factors relevant to the adoption and use of assistive technologies (table 1). Each participating organisation seeks to develop or change a service for older people living with multiple health and social care needs, with the help of technology. Some organisations recruited to date have focused on a particular technology (eg, telehealth monitoring for heart failure, Global Positioning System (GPS) monitoring for people with cognitive impairment), and seek to implement this in a target population; others seek to achieve a particular service goal (eg, reducing re-admission rates in high-risk patients), and are agnostic about which technology(ies) they will use. One has a very general goal—to become ‘digital by default’ (ie, maximise the use of technology where appropriate by investing in appropriate technologies and training and supporting staff and clients to use these). All participating organisations have signed up to an action research design in which ethnographic and other (mostly qualitative) data collected by or with our research team will feed into reflection, planning and action phases. Work in three of the five initial case studies started before the SCALS programme was funded, and has been able to continue as a result of that funding.

**Table 1:** Five organisational case studies in the SCALS (Studies in Co-creating Assisted Living Solutions) programme to date

Title	Organisation	Service challenge	Goal	Policy challenge	Technology(ies)
Case 1: Integrated care for people with multimorbidity	Clinical Commissioning Group in a deprived, multiethnic outer London borough (health sector)	To introduce and optimise home-based care for patients with complex needs	To prevent unnecessary hospital admission and reduce costs	Delivering integrated care that is truly ‘seamless’ to people with complex needs	For individuals: range of telecare devices (eg, alarms), mobility aids (eg, stair lifts), medical devices used at home (eg, oxygen) provided by health and social services and own adaptations of the home environment. For service providers: shared electronic care plans and ‘virtual ward’ database.
Case 2: Global Positioning System (GPS) ‘tagging’ for people with cognitive impairment	Council in a deprived, multiethnic inner London borough (social care sector)	To provide GPS devices to people with memory impairment (mild to moderate dementia)	To enable people to walk around their locality without fear of getting lost, and to reduce the risk of emergency callouts for lost citizens	Ethics and practicalities of ‘tagging’	Considering various tracking devices for example, ‘Buddi’ (http://www.buddi.co.uk)); ‘Vega watch (http://www.everon.fi/en/solutions/vega-gps-safety-solution-and-bracelet).
Case 3: Telehealth for heart failure	Acute trust and clinical commissioning group in south midlands university city (health sector)	To introduce and optimise telehealth services for patients with heart failure	To maximise quality and length of life and reduce emergency hospital admissions	Delivering care closer to home	Telehealth technologies (especially for weight and blood oxygen levels); video consultations via Skype or Facetime.
Case 4: Maximising uptake of telehealth and telecare	Clinical commissioning group in moderately deprived west midlands town (health sector)	To support delivery of telehealth and telecare through multiagency working	To improve the patient experience, reduce hospital admissions, save money	Delivering care closer to home	Range of telehealth and telecare technologies.
Case 5: Digital technology to reduce health and social care utilisation	Council in moderately deprived north-western town (social care sector)	To improve the experience of care and service efficiency	To empower citizens (including digital literacy), build cross-sector partnerships and share digital records	‘To prevent people becoming patients’	No specific technologies at this stage: considering a range of apps, software packages, devices. To date, one has been tried but rejected as unfit for purpose.

### Programme management and governance

The SCALS programme will be based at, and sponsored by, the University of Oxford. It will include academic partners at Queen Mary University of London and the University of Warwick; and National Health Service (NHS) and social care partners in participating sites across England.

The research will be organised as a series of organisational case studies as listed in table 1 (and with up to four additional case studies added later, taking account of the trade-off between depth and breadth). Each case study will be led by a locally based researcher. Meetings between research teams will occur at least 3-monthly by teleconference, and 6-monthly face-to-face to share emerging findings and develop the cross-case analysis.

The programme will be supported by an independently chaired, intersectoral steering group, with representation from the technology industry, health and social care services, policymakers (NHS England), people with assisted living needs, lay members and external academics. We anticipate that, as in our previous studies of technology development and adoption, this group will serve as a vibrant intersectoral discussion forum and a crucial, bidirectional conduit to national policy, the boards of NHS, local authority and industry partners and a link with front-line clinical and social services teams.

### Meso-level case studies

For each case study, we will use action research (the cycle of asking a question, collecting data, analysing data, initiating change and collecting more data to assess progress) to work with clinical and social care teams to address organisational aspects of addressing the service challenge, setting and meeting project goals, embedding particular technologies into particular microsystems and service models, and evaluating progress. In each case study, we will help the participating organisation(s) to apply the ARCHIE principles with a view to achieving patient centred, adaptive and co-created sociotechnical solutions (see above). We will also draw on Bate and Robert's experience-based co-design methodology (which requires the careful study of individual patient/client experience, for which ethnographic study is ideal), adapting this as needed to incorporate the introduction, embedding and adaptation of systems for supporting technology-mediated interactions between patients or clients, and health or social care professionals.^59^

We will combine this pragmatic and adaptive action research design with a more theory-driven approach aimed at collecting and analysing a dataset to produce a series of multilevel (micro–meso–macro) case studies, along with a cross-case analysis (see below). We have previously used action research to (simultaneously) facilitate and study the change process, and how conflict plays out and is managed in acute trusts, community trusts, and clinical commissioning groups, and have (in several different studies) successfully achieved the twin goals of action research: (1) local learning and change and (2) contribution to the wider knowledge base.^74–77^

### Micro-level ethnographies

The literature on the lived experience of ageing and assisted living includes numerous ethnographic studies by other research teams^20–22^ ^26^ ^37^ ^60–63^ ^65–67^ ^78^ as well as our own case series of 40 detailed ethnographies for the ATHENE study,^48^ ^49^ many of which have been published with participants’ consent (see http://www.atheneproject.org). In the SCALS programme, we will use in-depth ethnography of approximately 60–80 individuals (5–15 per case study) to inform the action research and meso-level theoretical analysis described above. These individuals will form the main (though not necessarily the only) group to inform the co-design process occurring at meso level.

Our sampling of individuals will be purposive so as to include people with *complex needs*, including (for example):

- People living with dementia (almost all of whom will have other physical, mental or emotional conditions), including the carer experience;
- People requiring personal and intimate care (eg, with washing, toileting);
- People with inexorably deteriorating and/or terminal conditions;
- People living with mental health problems as well as physical or cognitive impairments;
- People with potentially stigmatising conditions such as epilepsy, HIV, or alcohol or drug dependency along with other impairments;
- People living in profound poverty, unstable housing circumstances, adverse family circumstances (eg, no relatives or friends in touch) or uncertain citizenship status (eg, asylum seekers);
- People where there may be adult safeguarding concerns.

This sampling frame is deliberately constructed as a counterpoint to the ideal type ‘smart home’ resident (depicted as a confident, mentally capable owner–occupier with non-stigmatising illness, supportive family and no social problems^1^ ^79^).

Subject to ongoing consent or assent of the participant and their carer(s), we will conduct a series of ethnographic visits and use cultural probes (especially cameras for people to take pictures of what matters to them), home tours (in which the individual explains the different rooms in their home and how they are used) and ‘go-along interviews’ (accompanying the person on a journey of their choice) to capture their experience of multimorbidity and ageing from the perspective of both the index individual and their family and carer(s).^55^ ^57^ ^69^ ^80^ A particular focus will be the way in which participants and their carers attempt to use, adapt, combine and repurpose technologies in pragmatic ways within and outside the home to achieve what matters to them, and what kinds of constraints they encounter as they do so. Field notes, interview transcripts, photographic and other data will be combined using the narrative form to produce a rich descriptive case study of each participant. Interviews with general practitioners and/or social care staff looking after the person may be used to augment their personal narrative. These individual ethnographies will be theorised using phenomenology and structuration theory, paying particular attention to the material, symbolic and sociocultural aspects of technology use (and non-use), and fed into meso-level organisational learning and co-design.

### Macro-level analysis of wider context and public engagement

With two goals—gathering rich data on the wider context and maximising dissemination and application of our findings—in mind, we seek to build and maintain relationships with policymakers, industry and other key stakeholders nationally, both through our intersectoral steering group, and more widely.

As with the meso-level element of the programme, this element will be informed by the principles of action research. Thus, there will be cycles of developing relationships with national stakeholders, identifying problems and issues, feeding emerging findings back and discussing these, and exploring possible ways forward, workarounds or pragmatic compromises for policy, industry and the professions. In this way, project activities, such as steering group meetings, documents and so on, will be captured as research data and be included in our analysis, along with other data on the wider context (eg, blogs, press articles, industry documentation). Discussions with stakeholders will include both formal, audiotaped semistructured interviews and also ‘informal interviews’ undertaken in the process of setting up and steering the programme.^81^

We hope to identify, from the perspective of these stakeholders and with their active input to our deliberations, how to overcome policy, economic, technical and other barriers to delivering a robust, user-centred, sociotechnical microsystems approach to developing, customising, introducing and sustaining assisted living technologies for the various target groups in our case studies (some of whom, we anticipate, will be heavy users of health and social services, but hampered to a greater or lesser extent by poverty, low health literacy and multimorbidity). We do not anticipate that these deliberations will generate winning formulae or universal solutions. Indeed, we position our work against the *possibility* of such solutions.

Rather, we hope that multistakeholder input, both local and national, will produce a kind of pragmatic ‘muddling through’ that generates effective local solutions along with transferable insights about how the co-design process for technology-supported, person-focused health and social care services might be planned and operationalised. Local solutions, like the ARCHIE principles, will always require additional and ongoing work to adapt and refine them to particular organisations, individuals and settings.

One element of this macro-level component will be working with industry (as we have already begun to do) to help shift technology development and business models from ‘cathedral’ to ‘bazaar’ by encouraging the adoption of open standards to provide the necessary technical underpinnings for pragmatic, user-centred bricolage of a range of adaptable and interoperable devices by the potential user and his or her carers. A key enabler will be for industry to put in place the capacity to track the evolving relationship between devices and users’ needs and feed this back into design and development processes.

A second element, chiefly via their representation on our steering group, will be linking with national policymakers to consider how far our emerging findings make sense to those charged with developing and implementing policy on assisted living technologies in the UK—and what additional perspectives these policymakers bring that have not been (fully) addressed by local actors.

A third element will be working with policymakers and others (eg, professional and regulatory bodies) who set strategy and develop and implement standards and guidance for the use of assistive technologies. These will include NHS England, the National Information Board, Information Governance Alliance, Royal Colleges and defence societies. We will build our sample iteratively depending on how issues and policies emerge over the life of the programme. Among other things, we will want to probe whether the standard forms of evaluation and monitoring employed by regulatory agencies would be able to accommodate the pragmatic, ‘muddling through’ solutions we advocate.

A final and central element of this programme will be public engagement. We are grateful to the Wellcome Trust for a Public Engagement Award linked to the SCALS programme. In engaging citizens, we seek to do more than ‘disseminate findings’ to passive audiences. Rather, our goal is to use civic engagement—with third-sector organisations, schools, the press and other public fora—to help us address complex and contested moral questions about the role of technology in an ageing society—especially as it relates to the key policy questions illustrated by our case examples (column 5 in table 1), namely ‘tagging’, ‘integrated care’, ‘ageing in place’, ‘care closer to home’ and so on. These and other policy themes will guide our macro-level data collection and our public engagement efforts.

Our engagement with industry, policymakers and the lay public will also be used as an important source of data for the macro-level analysis. We will, for example, record, transcribe and analyse public debates on themes relating to ageing and new technologies. Press articles (both ‘lay and ‘technical’), workshop outputs (eg, flip chart paper), blogs, social media threads, online responses to our publications and so on will represent a rich corpus of material for analysing contemporary discourse on the key policy themes of interest to us. Using strong structuration theory as an analytic lens, we will nest our organisational case studies in this wider analysis of public, political and industry discourse.

### Integrating the dataset

This programme of work will generate a large amount of complex, multimodal data. It will be mostly qualitative (interviews, group discussions, observations, documents) but will also include aggregated quantitative data on the performance of local services (eg, access and uptake rates, biometric data, hospital admission rates), and technical and operational data (eg, technical design features, affordances, standard operating procedures). Furthermore, the nature of the data will probably change longitudinally over time as we learn what sources are most useful to our analysis. Managing and integrating these data will be a significant challenge but we anticipate that the diversity and richness of the data will ultimately allow us to produce a sophisticated higher order analysis and new theorisations.

As noted above, multimodal micro-level (ethnographic, interview, biometric, technical) data on individual participants will be drawn together into narrative summaries, and these summaries will be fed into the action research process in participating organisations. At the meso level, we will apply the principles of interpretive case study to produce narrative accounts of organisational development over time, focusing especially on how the organisation and its industry partners sought, and took account of, the user experience in evolving the design of the service and linked technologies, and how competing perspectives and conflicts of interest were surfaced and managed. These organisational case studies will be nested in the macro-level analysis of industry, policy, professional and citizen perspectives.

In developing the organisational case studies, we will initially draw on the work of Stake, who emphasised the importance of understanding the case for its own sake (‘what is going on here?’) rather than as an example of a particular theoretical phenomenon (What is this a case of?).^82^ ^83^ To understand the case for its own sake, richness—that is, granular depiction of real examples—is needed. As Weick has emphasised, richness has a number of *generative properties* including thick description, reflexive theorising and ‘conceptual slack’—openness to the many new explanations that emerge when contextual detail is added to the account.^84^ And as Stake has emphasised, it is important to tell the story ‘warts and all’:

We need to portray complexity. We need to convey holistic impression, the mood, even the mystery of the experience. The program staff or people in the community may be ‘uncertain’. The audiences should feel that uncertainty. More ambiguity rather than less may be needed in our reports. Oversimplification obfuscates. 85

This ‘warts and all’ perspective is important to tease out both the conflicts and the emerging synergies between different stakeholder organisations and different sectors.

The final stage in the analysis will be to synthesise findings across the sample of organisational case studies, and place these in the context of our macroanalysis of wider society. At this stage in the analysis, we will be looking for commonalities and contrasts across cases that will allow us to (1) consider the national ‘case’ of assisted living support in England and (2) at a more abstract level, make transferable statements about how organisations and networks can develop effective assisted living support. These statements will be such as to leave space for the variety of ‘bricolage’ solutions required to respond to the in situ specificities of particular organisations, individuals and settings. For the first of these tasks, we will still be asking ‘What is going on here?’ and producing a unique ‘n of 1’ case study account. But for the second, we will make more explicit use of strong structuration theory to consider how the macro context shapes, constrains and provides possibilities for the activity of organisations and networks (and, within those, individuals).

## Ethics and dissemination

### Ethics

The study of people with multimorbidity in their own homes, and the use of action research to support the introduction of technologies and services raises important and complex questions of research ethics, including consent, confidentiality and the nature of participation. Given that the study is funded through the Wellcome Trust's ‘Society and Ethics’ programme, all our research questions have an ethical dimension. Our theoretical perspective and analytic approach draws on the philosophy and theory of ethics (covering themes such as the ‘everyday ethics’ of what matters to people,^86^ the balance between autonomy and safety in caring for vulnerable older people,^87^ and the implications of a ‘panotpicon’ approach to digital surveillance).^71^

### Dissemination and projected outputs

An important feature of action research is that outputs and impacts are coproduced throughout the study.^58^ Rather than seeing the ‘outputs’ as a series of papers describing findings and the ‘impacts’ as how these findings are subsequently put into practice, action research *is itself* that putting-into-practice—and any publications are likely to be retrospective accounts of what changed during the study and how impact unfolded. In coproduced research, impacts are as much about (networked) relationships, ‘productive interactions’ and contributions to the *process* as they are about end outputs.^88^ ^89^ Two previous systematic reviews, one of community–campus partnerships and one of large-system change, identified *partnership synergy* and *distributed leadership* as key mechanisms through which positive change is generated in multistakeholder collaborations.^90^ ^91^

Hence, an important feature of the SCALS programme (as in our previous ATHENE work) will be the relationships that are built within and between the different partner organisations, both in the local study sites and nationally on the steering group, and the level of collective engagement by the multistakeholder collaboration in the unfolding projects.

Just as the ATHENE project produced detailed case studies of the individual experience of assisted living technologies, SCALS will produce richly described case studies of the organisational experience of trying to incorporate such technologies into *services*. We will work with our partner organisations to address how and to what extent these case studies will need to be adapted (eg, certain aspects redacted or fictionalised) for public consumption.

In principle, however, we seek to place detailed, granular descriptions of organisations’ efforts to deliver assisted living in the public domain. As Flybjerg has noted, ‘[A] scientific discipline without a large number of thoroughly executed case studies is a discipline without systematic production of exemplars, and … a discipline without exemplars is an ineffective one’.^92^ We believe the interdisciplinary field of assisted living will benefit greatly from our case studies, and furthermore, that our methodology may be taken up and applied by other intersectoral programmes to add more ‘thick descriptions’ to the currently sparse literature. Additional public outputs, supported by a Public Engagement Grant from the Wellcome Trust, will include promotion of debate, interaction with the media, and engagement with schools and other audiences.

## Conclusion

This paper has described a new (‘fourth generation’) approach to the study of assisted living technologies, characterised by interdisciplinarity, criticality, and a focus on complexity, recursivity and emergence. We seek to shift the debate from finding and scaling up universal technological solutions to exploring how good-enough sociotechnical arrangements can be co-created and sustained through human effort in the messy and contingent reality of local health and social care services.

Shifting from ‘designing particular technologies’ to ‘supporting adaptive bricolage for assisted living’ has significant implications for all stakeholders. For the technology industry, it poses challenges because it involves users in design and how it links with health and social care services. Currently in the UK, the business model is for industry to negotiate block contracts to supply a fixed menu of technologies (plus, usually, a maintenance contract). As well as shifting the design model from ‘cathedral’ to ‘bazaar’, allowing small, interoperable components to be purchased and combined in unique ways to address unique problems,^93^ we hypothesise that successful assisted living solutions will also depend on shifting the business model away from technology-focused contracting. For health and social care services, it poses questions about how to develop technology procurement policies and service delivery models that are capable of meeting users’ requirements, and being adapted as these requirements change. For policymakers, the challenge lies in devising strategies that will be successful in supporting adaptive evolution by other stakeholders, while also providing overall strategic direction.

The case studies that will come forth from SCALS will, we believe, provide a strong and credible evidence base for the technology industry, social care services and policymakers alike—though they will not produce a quick fix or universal winning formula. We also aim through SCALS to help in establishing mechanisms for the sustained cross-sector learning that is essential if assisted living programmes are going to deliver their promised benefits.
